# PRO: Environmental microbiological surveillance does support infection control in veterinary hospitals

**DOI:** 10.1093/jacamr/dlae113

**Published:** 2024-08-01

**Authors:** Dorina Timofte, Rosanne E Jepson

**Affiliations:** Department of Veterinary Anatomy, Physiology and Pathology, Institute of Infection, Veterinary and Ecological Sciences, Leahurst Campus, University of Liverpool, Chester High Road, Neston CH64 7TE, UK; Department of Clinical Science and Services, Royal Veterinary College, Hawkshead Lane, North Mymms, Hertfordshire AL9 7TA, UK

MDR healthcare-associated infections (HAIs) are a major challenge for human hospitals as they are associated with increased morbidity and mortality rates, as well as increased healthcare costs, and have become an increasing concern in veterinary settings.^1,2^ In particular, there is a perceived threat from a group of bacteria known as the ESKAPE organisms (*Enterococcus faecium*, *Staphylococcus aureus and Staphylococcus pseudintermedius*, *Klebsiella pneumoniae*, *Acinetobacter baumannii*, *Pseudomonas aeruginosa* and *Enterobacter* spp.) due to their tendency to be MDR and thereby ‘escape’ most antimicrobial agents.^3^ Importantly, the dual nature of the ESKAPE pathogens as environmental and commensal bacteria allows them to move freely between various niches (e.g. environmental, human and/or animal hosts), carrying and transferring antimicrobial resistance (AMR) genes and mobile genetic elements (MGEs). In this manuscript, the PRO position will argue that proactive targeted environmental surveillance that focuses on specific pathogens (e.g. ESKAPE organisms) is of benefit to clinicians and veterinary hospitals for guiding infection prevention and control (IPC) practices. In this approach, the results generated from environmental surveillance are serving as an early warning system that can be translated into change in IPC practice. The authors’ view is that this procedure is most relevant for the ESKAPE organisms, and the manuscript will discuss the PRO position with these organisms in mind. The PRO argument will focus on the targeted microbiological environmental surveillance, by which we refer to the selective screening of environmental surfaces for MDR organisms (MDROs), and more specifically screening for the ESKAPE organisms, in line with the CDC principle of ‘targeted sampling for defined purposes’ (https://www.cdc.gov/infection-control/hcp/environmental-control/environmental-sampling.html?CDC_AAref_Val=https://www.cdc.gov/infectioncontrol/guidelines/environmental/background/sampling.html). The current CDC guidance shows that microbiological environmental sampling is not discouraged when performed in accordance with defined protocols and carefully considered plans of action and control policy. This may include standardized methods for sample collection, the use of dedicated environmental sampling devices (e.g. moistened swabs, sponges, wipes, agar surfaces) available commercially and including chemicals to neutralize disinfectant residuals when expected to be present on surfaces being sampled. Furthermore, the CDC guidance shows that when sampling is conducted as part of an epidemiological investigation of a disease outbreak, identification of isolates to species level is mandatory, and characterization beyond the species level is preferred. Thus, molecular typing of clinical and environmental bacterial isolates plays an important role in epidemiological investigations of outbreaks or generally when attempting to determine the sources of infection.

Hospital settings are ideal for the development and selection of MDROs due to high antibiotic use and selective pressure. The development of large and specialized veterinary hospitals performing high-standard animal care, involving complex surgeries, intensive care facilities and greater reliance on antimicrobial therapy, has created similar conditions for the emergence of MDROs. Several studies have investigated the risk factors for animal patients becoming carriers of MDROs,^4–8^ whilst human and veterinary studies have shown that clinical environmental contamination with nosocomial pathogens can be an important reservoir for subsequent infection.^6,9^ Hence, rapid identification of the main reservoirs and understanding the routes of transmission for these pathogens are key to preventing HAIs in human and veterinary patients.

Despite the commonalities between the human and veterinary settings, it is not always feasible to directly transfer the same preventative measures in veterinary hospitals and indeed within the veterinary sector, differences are likely to be appreciated between small-animal and equine facilities. Hence, we need to generate veterinary-specific data to enable the development of evidence-based infection control policies and thus protect patient health and reduce the risk of zoonotic transmission to veterinary staff, owners and the wider community. Early identification of infections potentially linked to an environmental source is key to enabling rapid and effective interventions to limit transmission and outbreak development.

In the last decade, several important publications have provided key guidance for establishing veterinary infection control programmes in veterinary settings.^10,11^ These resources have proved to be important pillars for guiding veterinary infection control practices addressing aspects such as personal protective equipment, cleaning and disinfection, hand hygiene, staff education and training. However, the major issues of surveillance and environmental containment of MDROs in veterinary settings have not been addressed. As the evidence accumulates that common clones of ESKAPE are now circulating in human and veterinary patients,^12^ guidance is available for the management of MDROs. For instance, the CDC has introduced guidance, ‘Management of MDROs In Healthcare Settings’, in 2006 (https://www.cdc.gov/infection-control/hcp/mdro-management/index.html), whilst in the UK, a Joint Working Party (JWP) group of infectious diseases specialists and scientists has developed NICE-accredited guidelines for prevention and control of MDROs based on systematic reviews of peer-reviewed published research and expert opinions.^13^ A recent publication (Fahy *et al.*^14^) refers to the lack of official guidance on preventative MDRO environmental screening as the ‘elephant in the room’ and concludes that more needs to be done to implement microbiological screening for MDROs in the hospital environment, in order to identify and eliminate environmental reservoirs. There is wide agreement in the veterinary community that infection control programmes for veterinary settings should be tailored to each facility, reflecting the patient and pathogen risks, hospital facilities and antimicrobial use policies.^11^

## The veterinary hospital surface environment is likely to play a role in MDRO transmission

Early human infection control studies published before the 1980s suggested that hospital environmental surface contamination may play a negligible role in the transmission of HAIs.^9^ However, accumulating scientific evidence demonstrates an increased risk for other patients when hospital surface environments surrounding patients with MDRO infections are heavily contaminated.^14^ The role of the environment in transmission of several key pathogens has been reported, with an estimation that up to 20% of HAIs occurring in ICUs are due to environmental contamination.^15^ Weber *et al.*^15^ have reviewed the current methodology for microbiological sampling and highlight studies that have demonstrated that the proportion of surface contamination in rooms of colonized or infected patients can vary from 1%–27% for MRSA, 7%–29% or even 60%–70% for patients with multiple site VRE colonization, and 3%–50% for *Acinetobacter* spp. Similar data demonstrating the degree of microbiological surface contamination with MDROs in the veterinary hospital environment are lacking. However, several studies have shown that veterinary hospital surfaces are often contaminated with enterococci, ESBL-producing Enterobacterales and MRSA/methicillin-resistant *S. pseudintermedius* (MRSP).^4–6,16–18^ We have reported on the likely transmission of ESBL-producing *Escherichia coli* ST410 (a newly emerging MDR clone^19^) between the hospital environment and a canine patient, where targeted MDRO surveillance led to the detection of ESBL-producing *E. coli* ST410 from patient surgical wounds and the surrounding environment, including the ultrasound table and various areas of the ward where the dog was hospitalized (e.g. the door handle, the fridge handle and the computer keyboard).^20^ Although the direction of transmission was not determined, a pattern of hand cross-transmission has emerged, which could lead to further undetected dissemination. These findings were followed by closely followed by repeated auditing of cleaning and disinfection protocols, and reinforcing hand hygiene, which led to a lack of pathogen detection in the environmental during subsequent sampling.

JWP guidelines recommend microbiological environmental screening where there is unexplained transmission of MDROs or there is a possible common environmental source of an outbreak, with sites that are likely to be most relevant for cross-transmission selected for screening.^13^ We would add that targeted MDRO surveillance of high-touch surfaces also provides an opportunity to feed results back to staff, thereby increasing awareness of the importance of staff hand hygiene and clinical management of patients with MDRO infections. A recent observational study in Switzerland has shown overall poor (32%) hand-hygiene compliance in small animal clinics in practices, and concluded that educational interventions similar to those established in the human sector (e.g. individual training, lectures, give feedback to users, implement reminders) are also urgently needed in veterinary settings.^21^

## Targeted MDRO screening informs local and wider surveillance efforts

We fully agree with Burgess *et al*.^10^ that ‘*one cannot manage what one does not measure*’, therefore targeted microbiological surveillance provides the opportunity for data collection necessary to establish a baseline of MDRO environmental contamination at each veterinary facility. MDROs can spread long before being detected; consequently, surveillance can inform future interventions to limit their spread. Although the prevalence of MDROs is likely to vary between different veterinary settings, their distribution in veterinary hospital environments and their contribution to HAIs are largely unknown. Therefore, we need to generate veterinary-specific MDRO surveillance data. Consequently, in order to improve our knowledge of the molecular epidemiology of MDROs in the veterinary hospitals at the University of Liverpool (Equine and Small Animal) Hospitals, we performed a 6 month pilot project to investigate the rate of Gram-negative (GN) MDRO introductions in veterinary hospital environments and collected faecal samples from patients admitted to ICUs and the environmental surroundings in the first 48 h from admission. The study found that important ESKAPE pathogens including *P. aeruginosa* and *Enterobacter cloacae* (22% each), *K. pneumoniae* (15%) and *A. baumannii* (14%) were the most prevalent GN MDROs circulating in both the small-animal and equine hospitals.^22^ Molecular typing revealed that bacterial clones with identical resistome profiles were associated with a particular setting (e.g. some *Enterobacter* spp. were only found in the equine hospital, whilst *K. pneumoniae* types were only found in the small-animal hospital) and others (e.g. *A. baumannii* and *P. aeruginosa*) were shared between the two hospitals. These findings led to re-enforcing hand-hygiene measures and footwear protocols for staff and students to prevent inter-hospital spread of these organisms. In addition, despite the lack of carbapenem use, we have detected isolates carrying genes conferring resistance to carbapenems, such as *Acinetobacter* spp. harbouring *bla*_OXA-23_ and *E. coli* having *bla*_OXA-48_ resistance genes, within the hospital environment.^22^ These findings warrant increased surveillance in order to monitor the emergence of new MDRO strains in the veterinary hospital environment before their spread is more difficult to contain. Thus, prospective longitudinal rather than cross-sectional studies may better clarify the role played by the environment in transmission, as demonstrated by Dazio *et al.*, who identified an unexpectedly high rate of acquisition of MDR Enterobacterales during hospitalization in veterinary clinics in Switzerland.^7^

Targeted microbiological environmental surveillance as part of the IPC strategies for veterinary hospitals are rarely implemented due to concerns over costs and benefits; however, we need to also consider that the lack of surveillance may prove more costly in the longer term. To date, no studies in veterinary medicine have specifically evaluated the cost–benefit analysis of targeted microbiological environmental surveillance as part of infection protection policy and prevention of HAI. However, studies have documented the potential expense of nosocomial outbreak situations, for example in relation to an MDR *Salmonella* outbreak in a veterinary teaching hospital, which led to closure of the institution and substantial financial impact (US$4.12 million).^23^ Where targeted microbiological environmental surveillance is performed, this provides the potential to act in terms of environmental hygiene prior to a potential outbreak situation. Further work is needed in the veterinary sector to fully understand the financial implications of targeted MDRO surveillance in comparison with the financial burden to both hospital and client of HAI and outbreak scenarios.

## Clinical MDRO surveillance is slow and less sensitive

Infection control guidelines include passive disease monitoring of diagnostic data as a means of accessible (and inexpensive) surveillance. Human JWP guidelines indicate that passive surveillance of clinical infections is relatively insensitive and generally slow in identifying outbreaks of MDR GN infections.^13^ Furthermore, whilst some infections linked to unusual environmental bacteria (e.g. *Stenotrophomonas maltophilia*, *Achromobacter xylosoxidans*) may be easily observed, HAIs linked to more common bacterial pathogens, such as Enterobacterales, can often go unnoticed without comprehensive surveillance. It may therefore be fair to assume that in the absence of targeted MDRO surveillance in both clinical infections and the hospital environment, a number of veterinary HAIs and even outbreak situations go undetected. At the same time, clinical microbiology laboratories may play an important role in monitoring unusual pathogens or isolation patterns, thereby supporting MDRO surveillance efforts.

## Targeted MDRO surveillance—just another tool in the box

Although cleaning and disinfection are key elements of infection control programmes, this cannot always eliminate the pathogens of concern. Both human and veterinary studies have shown that the cleaning can often be suboptimal or that pathogens have developed ways to withstand the chemical germicide effects (e.g. biofilm formation, developing resistance to disinfectants).^24–26^ An excellent example of such a pathogen is *A. baumannii*, strains of which have developed augmented resistance to multiple antimicrobials and disinfectants, and have produced biofilm, which increases its survival in hospital environments.^27^ We have also encountered persistent MRSA in the equine hospital environment linked to the presence of the biofilm-related genes *icaA* and *icaD*, leading to persistence and resistance to disinfectant action despite repeated targeted deep cleaning.^28^ In addition, recent studies have shown that compliance with IPC standards can be poor in veterinary settings^29^ with one prospective study concluding that IPC standards implemented in veterinary practices in Switzerland are variable and that this was associated with extensive environmental MDRO contamination.^30^ On the other hand, the increasing number of publications linking environmental contamination to increased risk of HAIs, show that even when the best infection control practices are routinely employed, transmission of MDROs continues to occur in healthcare facilities worldwide.^1,31^ Hence, it is likely that the role of the environment in the acquisition of HAIs is still underestimated and that additional interventions (including environmental sampling) may be required to understand the extent and the role played by environmental contamination, in order to augment existing IPC measures.

In conclusion, the evidence shows that targeted MDRO environmental surveillance has proved useful for identification of environmental reservoirs and outbreak investigation, providing early warnings and opportunities for intervention to prevent further dissemination. Surveillance is strengthened when a broader approach, linking environmental MDRO surveillance to clinical cases, is implemented. This approach can be supplemented by developing protocols for saving MDRO isolates from both sources for subsequent epidemiological investigation. In all cases, the methods used for targeted microbiological sampling, the interpretation of results and the subsequent interventions need to be clearly defined and ideally standardized; thus, further research is needed to establish the evidence base necessary to address these gaps in our knowledge within veterinary settings.

With the slow progress in the discovery of new antimicrobials, targeted environmental surveillance could play an important role as part of a multifaceted approach to antimicrobial stewardship by monitoring the burden of MDROs in the environment, providing opportunities for interventions to reduce the risk of transmission to patients, thereby reducing the need to use antibiotics.
